# Biodegradation Behavior of Poly(Butylene Adipate-Co-Terephthalate) (PBAT), Poly(Lactic Acid) (PLA), and Their Blend in Freshwater with Sediment

**DOI:** 10.3390/molecules25173946

**Published:** 2020-08-29

**Authors:** Ye Fu, Gang Wu, Xinchao Bian, Jianbing Zeng, Yunxuan Weng

**Affiliations:** 1 Beijing Key Laboratory of Quality Evaluation Technology for Hygiene and Safety of Plastics, College of Chemistry and Materials Engineering, Beijing Technology and Business University, Beijing 100037, China; fuye@btbu.edu.cn; 2 Collaborative Innovation Center for Eco-Friendly and Fire-Safety Polymeric Materials (MoE), National Engineering Laboratory of Eco-Friendly Polymeric Materials (Sichuan), State Key Laboratory of Polymer Materials Engineering, College of Chemistry, Sichuan University, Chengdu 610064, China; gangwu@scu.edu.cn; 3 Key Laboratory of Polymer Ecomaterials, Changchun Institute of Applied Chemistry, Chinese Academy of Sciences, Changchun 130022, China; xcbian@ciac.ac.cn; 4 Chongqing Key Laboratory of Soft-Matter Material Chemistry and Function Manufacturing, School of Chemistry and Chemical Engineering, Southwest University, Chongqing 400715, China; jbzeng@swu.edu.cn

**Keywords:** PBAT, PLA, freshwater sediment, degradation

## Abstract

Poly(butylene adipate-co-terephthalate) (PBAT) and poly(lactic acid) (PLA) are well-known biodegadable polyesters due to their outstanding performance. The biodegradation behavior of PLA/PBAT blends in freshwater with sediment is investigated in this study by analyzing the appearance, chemical structure and aggregation structure of their degraded residues via SEM, TG, DSC, gel permeation chromatography (GPC) and XPS. The effect of aggregation structure, hydrophilia and biodegradation mechanisms of PBAT and PLA on the biodegradability of PLA/PBAT blends is illuminated in this work. After biodegradation, the butylene terephthalate unit in the molecular structure of the components and the molecular weight of PLA/PBAT blends decreased, while the content of C-O bond in the composites increased, indicating that the samples indeed degraded. After 24 months of degradation, the increase in the relative peak area proportion of C-O to C=O in PLA/PBAT-25, PLA/PBAT-50 and PLA/PBAT-75 was 62%, 46% and 68%, respectively. The biodegradation rates of PBAT and PLA in the PLA/PBAT blend were lower than those for the respective single polymers.

## 1. Introduction

As widely used biodegradable polymer materials, PLA and PBAT are applied to prevent the continual worsening of global ‘white pollution’. PLA is chemically converted and polymerized with lactic acid which is produced from various starches, sugars, and other biomass materials through biological fermentation [1,2]. The bio-based PLA can be thermo-plastically processed into highly transparent products to replace the conventional disposable products and inhibit the increasing scarcity of petroleum resources [3,4,5]. Due to the flexibility and high elongation at break, PBAT and its composite are widely used as biodegradable blown film products. Compositing PLA and PBAT is an effective method to combine and improve their comprehensive performance. The blending process, compatibility, crystallization, rheological properties and mechanical properties of PLA/PBAT blends are studied to illustrate the toughening of PLA with PBAT by modifying the brittleness and crystallization of PLA [6,7,8]. Compounding PLA and PBAT gives immiscible blends, and the final properties of the blends are highly dependent on the intrinsic properties of the blend components and blend morphology. The morphology of polymer blends depends on the interfacial tensions between the polymer phases which can be significantly improved by chain extender and compatilizer [9,10]. Wang et al. developed a high-toughness PLA/PBAT blend by improving the mechanical properties and rheological behaviors via in situ reactive compatibilization, in which a large number of branched copolymers formed by the reaction between epoxy groups of functional compatibilizer and the terminal carboxyl and hydroxyl groups of PLA and PBAT [11]. Another approach to control the morphology of polymer blends with emulsion type microstructure, while improving the properties of the blend, is the incorporation of nanoparticles. The interaction between nanoparticles and the component as well as their prevention of polymer droplet coalescence at the interface affects the morphology stabilization and rheological properties of PLA/PBAT blends [12]. Jiang et al. fabricated a layered structure PLA/PBAT composite with the skin layer exhibiting higher chain alignment and conformational ordering and higher mechanical properties than the core layer by doping rigid nanoparticles, i.e., montmorillonite clay or nanosized precipitated calcium carbonate [13]. Tubular nanoclay in the composite can agglomerate into bundles that were positioned parallel to the interface, blocking the chain entanglements and contributing to a weakening of the interfacial adhesion [14]. Shankar et al. used three different types of zinc oxide nanoparticles (ZnONP) to reinforce PLA/PBAT blend films. The incorporation of ZnONP leads to improved optical and mechanical properties as well as strong antibacterial activity of the composite [15].

**Figure 1:**
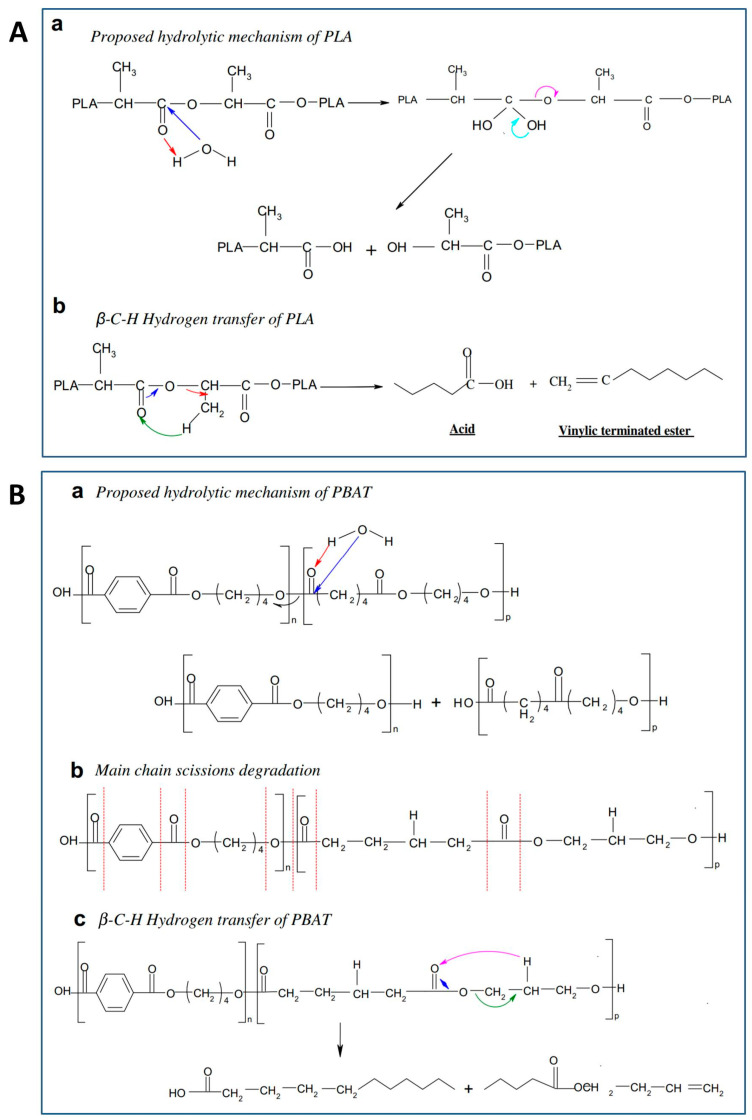
Schematics of hydrolytic degradation mechanisms of the most widespread biodegradable polyesters: (**A**) poly-lactic acid (PLA), (**B**) poly-butylene adipate-co-terephthalate (PBAT) [21]. (reproduced with permission from Elsevier).

PLA and PBAT can degrade into carbon dioxide, water and other small molecules under soil and compost conditions with the influences of heat, water, oxygen, organisms and enzymes [16,17,18]. The decomposion of PLA macromolecules mainly occurs at the end of the molecules by a zipper-like undoing mechanism, the process of which is influenced by the molecular weight, crystallinity, purity and stabilizers of PLA [19,20]. PLA can be biologically decomposed into carbon dioxide and water under composting condition. Hydrolytic chain scission and main chain scission are two common degradation pathways of PLA (shown in Figure 1A) [21]. The degradation determines a dramatic molecular weight reduction and can be easily monitored by spectroscopic measurement of –OH and –COOH groups, since the degradation products are hydroxyl- or carboxyl-terminated PLA [22]. However, the biodegradation rate of PLA under soil condition and water condition is relatively slow [23,24]. Different from PLA, PBAT is an aliphatic–aromatic biodegradable polyester produced from fossil sources. The aromatic fraction provides excellent physical properties, whereas aliphatic chains promote its degradation in several conditions, including soil degradation without the need for temperature control [25]. The biodegradation of PBAT can be regarded as the hydrolysis under the effect of microbial enzymes, during which the BA structure with non-crystalline portion degrades faster than the BT structure with a crystalline portion [26]. PBAT undergoes hydrolytic degradation owing to the cleavage of ester linkages and the reaction between water and the carbonyl groups located in the proximity of the benzene rings (Figure 1B). β-C-H hydrogen transfer reactions are supposed to occur randomly, even in the PBAT backbone [21]. The packaging applications of PLA (the current most promising bio-based polymer) are seriously restricted by the low toughness caused by its inherent brittleness and low ductility, and PBAT is generally added to make the disposable products degrade quickly enough to meet the home compostability criteria.

The biodegradation behavior of PLA/PBAT blends under soil condition has been illuminated by the authors. It has been found that the degradation rates of PBAT and PLA in PLA/PBAT blend under soil condition are different from the degradation rates of the respective single polymers [27]. Palsikowski et al. compatibilized PLA/PBAT blend with chain extender and evaluated its biodegradability in soil. The chain extender functional groups reacted with the groups generated during the polymers’ degradation in a competitive effect with molecular weight reduction, causing biodegradation delay. The blends show intermediate behavior compared to the original polymers [28]. Ajji et al. analyzed the ester bond change in the PLA/PBAT blends after biodegradation and revealed the selectivity of composting to degrade the PLA phase in PLA/PBAT blends to a higher extent and form a porous 3D network. Formation of these networks explains the retarded disintegration of the blends [29]. The authors studied the biodegradation behavior of PLA, PBAT and their blends under digested sludge conditions and found that the degradation rate of PLA obtained from respiratory test was higher than that of PBAT [30]. In the research of the degradation behavior of PLA, PBAT and poly(aspartic acid-co-lactide) (PAL) blend films in 40 °C phosphate buffer, Oyama et al. found that the degradation rate of blends was not only significantly affected by the PAL content and composition but also promoted by the degradation products of PLA [31]. The increasing emphasis on preventing water pollution has aroused the exploration of the degradation behavior of biodegradable materials under aqueous conditions. However, little research has been published on the biodegradation behavior of these materials in water.

In this work, the biodegradation behavior of PLA/PBAT blends in freshwater with sediment was investigated by analyzing the appearance, chemical structure and aggregation structure of their degraded residues. The effect of aggregation structure, hydrophilia and biodegradation mechanisms of PBAT and PLA on the biodegradability of PLA/PBAT blends was illuminated. Gel permeation chromatography (GPC) and XPS were used to characterize the change in film sample molecular weight and element state, through which we can analyze the molecular structure of the components and PLA/PBAT blends after the incubation in freshwater with sediment. This exploration of the degradation behavior of PLA/PBAT blend films can provide a theoretical foundation for the design and analysis of biodegradable materials which offers a prevention method of water pollution.

## 2. Results and Discussion

**Figure 2:**
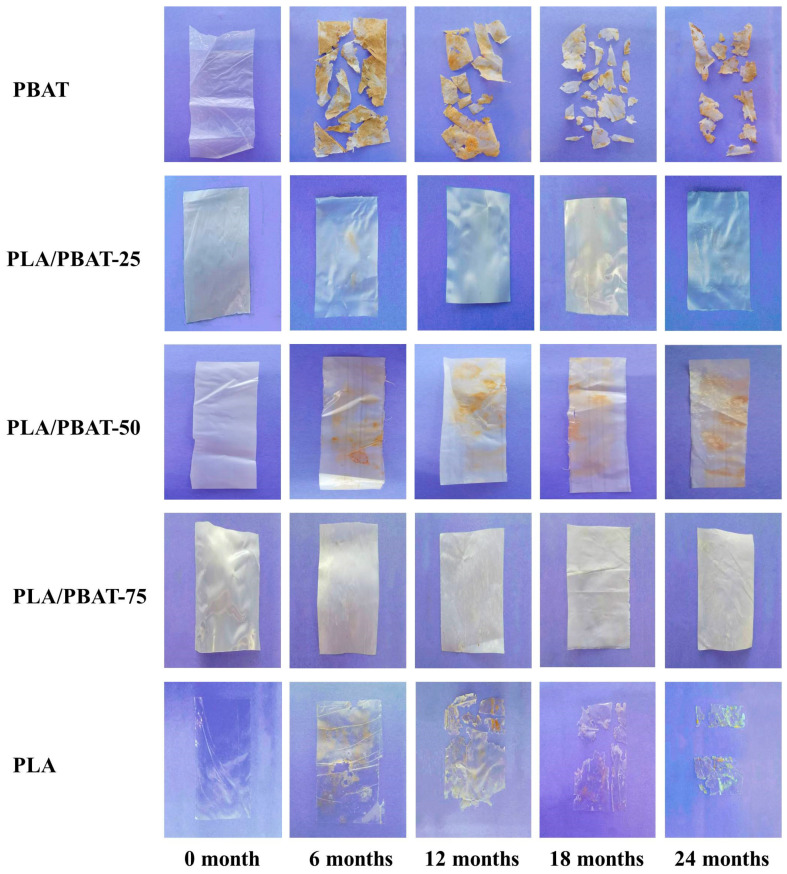
The appearance changes of PBAT, PLA and PLA/PBAT films with different incubation times in the freshwater sediment.

The appearance changes of the polymer films after months of incubation in freshwater sediment are shown in Figure 2. The PBAT, PLA/PBAT, and PLA samples were gradually degraded with the increase in incubation time. The thickness of PBAT and PLA films before degradation is 0.076 ± 0.016 and 0.049 ± 0.010 mm. After 6 months, PBAT film turns brown and breaks, and 24 months later only a small amount of residual fragments remain. For the PLA sample, film transparency reduces and erosion holes appear after 6 months of incubation; 24 months later, the film becomes brittle and breaks into residual fragments. For the PLA/PBAT samples, after 6 months, the films turn white and become brittle. The thickness of PLA/PBAT-25, PLA/PBAT-50 and PLA/PBAT-75 are 0.080 ± 0.023, 0.097 ± 0.028 and 0.115 ± 0.035 mm, which, respectively, increases to 0.133 ± 0.040, 0.116 ± 0.038 and 0.121 ± 0.038 mm after 24 months of degradation. The films of PLA/PBAT show some internal defects and cavities after incubating for 24 months, which indicates the degradation of composites. It is obvious from the evidence that PBAT and PLA samples in the freshwater with sediment perform faster degradation rate than the PLA/PBAT samples.

The water contact angles of PBAT, PLA/PBAT-25, PLA/PBAT-50, PLA/PBAT-75 and PLA are 86.2° ± 3.9°, 81.7° ± 3.0°, 75.6° ± 3.1°,67.3° ± 2.8° and 56° ± 2.5°, respectively (Figure S1). The contact angle of PLA/PBAT is reduced with the enhanced content of more hydrophilic PLA in the composite. The hydrophilia of composite is an important influence on the hydrolysis rate of materials. The more hydrophilic the composite is, the stronger the adsorption capacity of water molecules on the material surface is, which leads to a higher hydrolysis rate as a result of the higher attack efficiency of the ester bond on the material molecular chain by the hydroxyl group in the water molecule.

**Figure 3:**
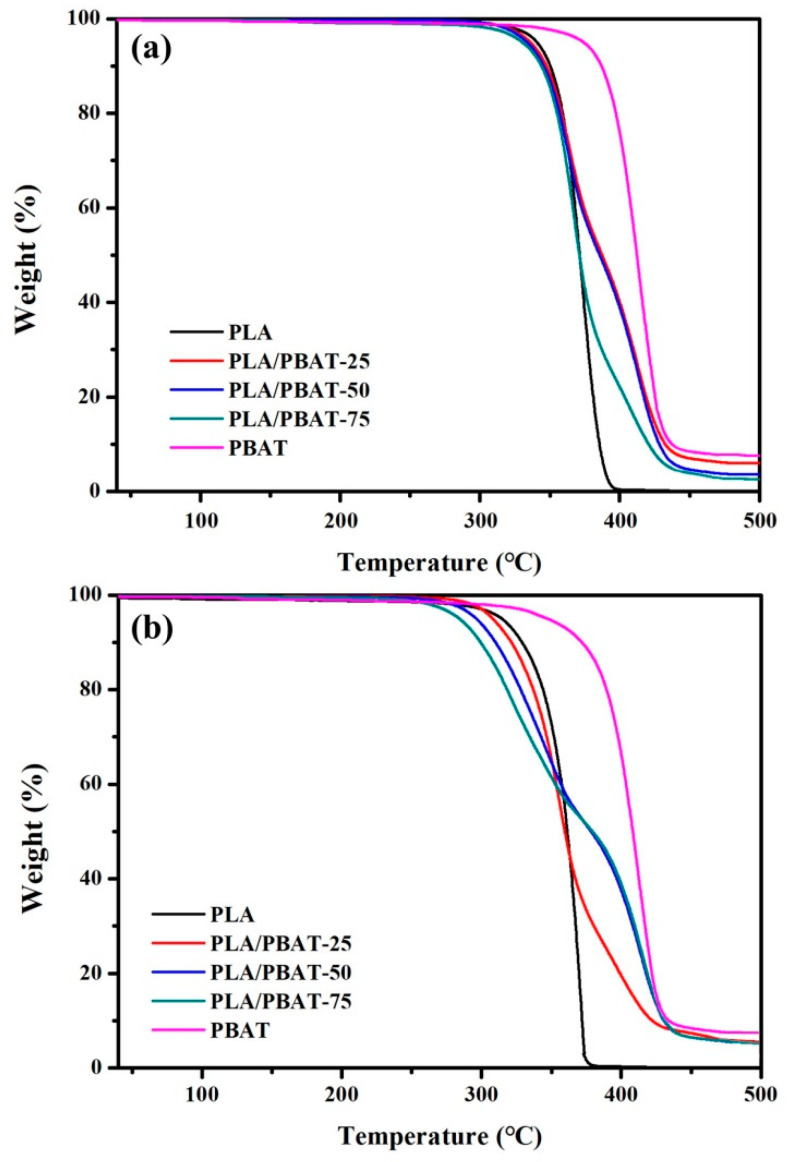
TG curves of PLA/PBAT composite films (**a**) before and (**b**) after 24 months of degradation.

The thermal stability and thermal decomposition of PLA/PBAT composites was evaluated by measuring the onset degradation temperature of 5% mass loss, peak degradation temperature and ash content with TGA at various temperatures. The TGA curves of composite films before and after 24 months of biodegradation are shown in Figure 3. The peak degradation temperature of PLA is reduced to 330–336 °C by the inclusion of PBAT. After 24 months of biodegradation, the onset degradation temperature of 5% mass loss and peak degradation temperature of composites shifts to a lower temperature, which is due to the decreased molecular weight after degradation. The degradation temperature of PBAT after degradation migrates to lower temperature not as significantly as PLA, which can be attributed to the faster molecular chain fracture of PLA. The onset degradation temperature of 5% mass loss, 50% mass loss temperature and peak degradation temperature of PLA, PLA/PBAT, and PBAT samples before and after months of degradation are listed in Table S1. The TG temperature of the composite after degradation is basically the same as PLA and PBAT curves, which indicates the degradation processes of the components in PLA/PBAT blend still follow that of the respective single polymers.

**Figure 4:**
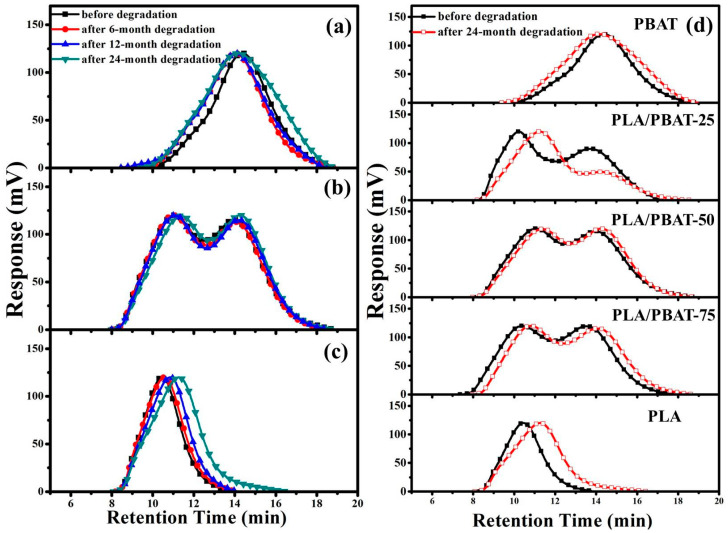
Gel permeation chromatography (GPC) curves of (**a**) PBAT, (**b**) PLA/PBAT-50, (**c**) PLA before and after degradation for different time, and (**d**) PLA/PBAT composite with different PLA content before and after 24 months of degradation.

The molecular weight of PLA, PLA/PBAT and PBAT before and after incubation was analyzed to demonstrate the degradation of composite films. The GPC curves of PBAT, PLA/PBAT-50 and PBAT of before and after 6, 12, and 24 months of degradation are shown in Figure 4a–c. The full width at half maximum (FWHM) of PBAT and PLA curves are gradually broadened after the degradation, while the peak positions of both shift to longer retention time. This indicates that the molecular weight decreases and the molecular weight distribution increases after the degradation. In the GPC curves of PLA/PBAT-50, the peak position attributed to PLA shifts more significantly than that of PBAT, which reflects the faster molecular weight reduction of PLA. The GPC curves of PLA/PBAT composite films with different PLA content before and after 24 months of degradation (Figure 4d) show that the degradation processes of the components in blend still follow that of the respective single polymers. The relative peak intensity of PBAT in PLA/PBAT-25 reduced significantly after 24 months of degradation, which indicates the faster degradation of PBAT than that of PLA in the composite. The largest peak shift and the highest FWHM increase for PLA/PBAT-25 among the three composites is attributed to the decreased molecular weight and broadened molecular weight distribution illustrates its highest degradation degree. The quite small peak shift range of PLA/PBAT-50 demonstrates the lowest degradation degree of composite, which is consistent with the result of the highest crystallinity of the blend. The slightly offset nature of the PLA/PBAT-75 curve after 24 months of degradation indicates the reduction in components’ molecular weight. The inclusion of PBAT affects the molecular weight retention rate of PLA, which demonstrates that the PBAT content in the composite is an important factor of the PLA/PBAT composite degradation rate.

**Figure 5:**
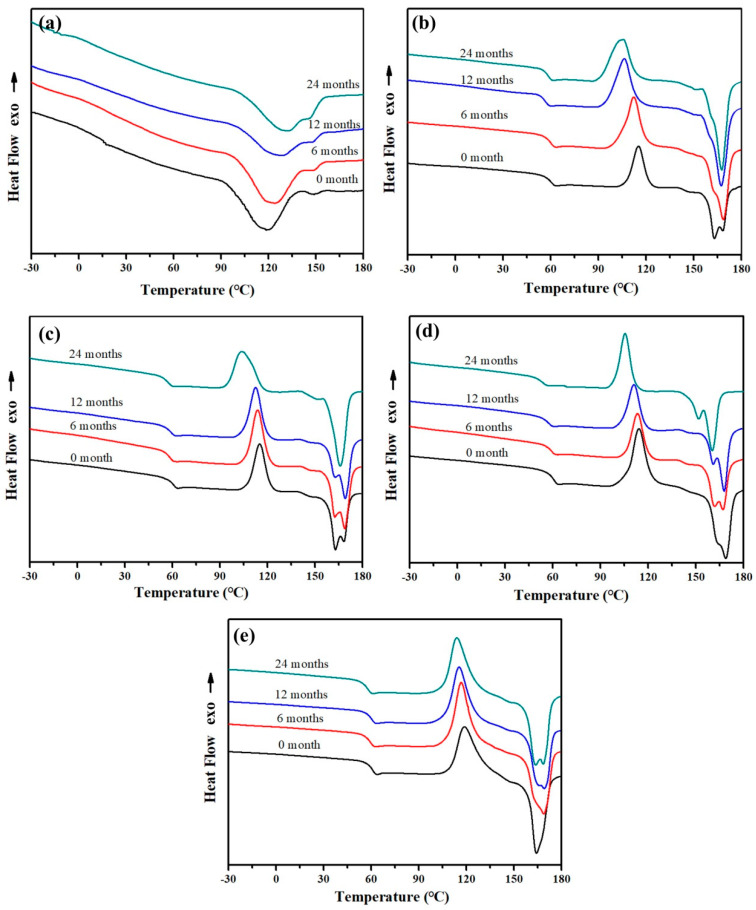
DSC melting traces of (**a**) PBAT, (**b**) PLA/PBAT-25, (**c**) PLA/PBAT-50, (**d**) PLA/PBAT-75 and (**e**) PLA before and after degradation for 6 months, 12 months, 24 months.

The aggregation structure of PBAT and PLA blends is illuminated with DSC analysis. The amorphous regions in a semi-crystalline biodegradable polyester are usually more susceptible to microorganisms and enzymes than the crystalline regions. The less compact structures would eventually facilitate the biodegradation and hydrolysis of the amorphous regions prior to the crystalline regions. The butylene adipate (BA) units and butylene terephthalate (BT) units in PBAT share a common crystal lattice by adjusting the soft BA units into the rigid BT crystal lattice to form a co-crystallization or a mixed-crystallization structure. The biodegradation and hydrolysis in the vulnerable aliphatic BA units leads to a better crystalline structure with fewer defects, which is reflected in the increased melting temperature (Figure 5a). The $T_{g}$ and $T_{m}$ of PLA before biodegradation are 61 °C and 165 °C. After 24 months of degradation, the $T_{g}$ of PLA decreases to 56 °C due to the free movement of polymer segments, while the melting peak splits into two peaks, which is a result of the melting of imperfect crystalline regions formed by the fractured molecules and the melting of secondary crystals followed by recrystallization. In PLA curves, the typical cold crystallization peak at 120 °C associated with the rearrangement of amorphous phase into new crystalline regions shifts to the lower temperature after the degradation.

With the increased addition of PLA in the composite, the $T_{g}$ of PLA in the composites after 24 months of degradation is reduced from 58 to 54 °C. Meanwhile, the relative peak area of the melting peak of secondary crystals enlarges with the increased PLA content. The crystalline fraction $X_{c}$ (%) of PLA/PBAT can be calculated by the equation:

$$X_{c}=\frac{\Delta H_{f}-\Delta H_{c}}{w_{PLA}\cdot \Delta H_{f,PLA}^{0}+w_{PBAT}\cdot \Delta H_{f,PBAT}^{0}}\times 100\%$$

where $\Delta H_{f}$ and $\Delta H_{c}$ are, respectively, the enthalpy of fusion and cold crystallization of the composites. $\Delta H_{f,PLA}^{0}$ = 93 J/g and $\Delta H_{f,PBAT}^{0}$ = 114 J/g are taken as the heat of fusion of an infinitely thick PLA and PBAT crystal, respectively [32,33]. $w_{PLA}$ and $w_{PBAT}$ are the PLA and PBAT content in the composite. The $X_{c}$ of PLA/PBAT with different component proportion before and after degradation are listed in Table 1. The crystallinity of PLA/PBAT composites increases with the degradation time, which is a result of the faster biodegradation of amorphous regions by microorganisms and enzymes.

The chemical compositions of the surfaces of PLA/PBAT composite films are determined by XPS. The element content ratio of O/C shows a significant increase after degradation (listed in Table 2). The increment of the O/C content ratio in PBAT is 273.3% after 12 months of degradation and 289.4% after 24 months of degradation, while the increment of O/C content ratio in PLA is 85.3% after 12 months of degradation and 186.5% after 24 months of degradation, which indicates that the hydrolysis adequacy and rate of ester bond in PBAT are both higher than in PLA. The increment of O/C content ratio in PLA/PBAT composite films represents a strong relativity to the components. Elevated PLA content in the composite leads to a higher increase in the O/C content ratio after degradation.

**Figure 6:**
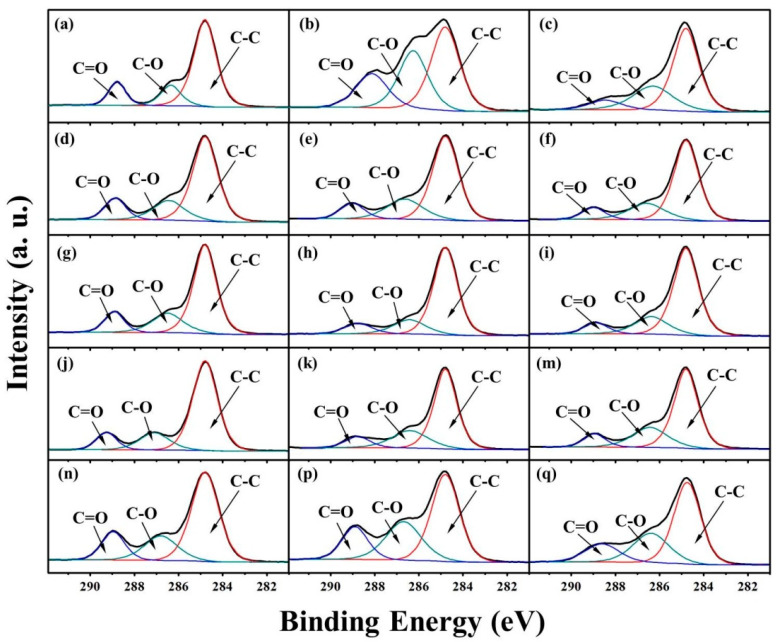
XPS C1s core-level spectra of (**a**–**c**) PBAT, (**d**–**f**) PLA/PBAT-25, (**g**–**i**) PLA/PBAT-50, (**j**,**k**,**m**) PLA/PBAT-75 and (**n**,**p**,**q**) PLA before and after degradation for 12 and 24 months.

Figure 6 shows the C 1s core-level spectra of PBAT, PLA/PBAT-25, PLA/PBAT-50, PLA/PBAT-75, and PLA before and after 12 and 24 months of degradation. The C 1s core-level spectra can be curve-fitted into three peak components at BEs of about 284.8, 286.5, and 288.9 eV, attributable to the C-C, C-O, and C=O species, respectively. The peak component for C=O is due to the ester linkages and the hydrolysate carboxyl in the biodegradable polyester. The peak component for C-O, meanwhile, is contributed by both the ester linkages and its hydrolysate. The hydrolytic degradation in blends owing to the cleavage of ester linkages and the reaction between water and the carbonyl groups forms the degradation products of hydroxyl- or carboxyl-terminated PLA and PBAT. The relative peak area proportion of C-O to C=O of PBAT, PLA and PLA/PBAT composites rises after the incubation in freshwater sediment, which reveals the degradation of polymer films. The distinction between the characteristic peak intensity of PBAT before and after degradation is obvious, with the relative peak area proportion of C-O to C=O increasing from 1.06 to 3.02. The relative peak area proportion of C-O to C=O in PLA is 1.27, 1.63 and 1.71 before and after degradation for 12 months and 24 months, respectively. After 24 months of degradation, the increase in relative peak area proportion of C-O to C=O in PLA/PBAT-25, PLA/PBAT-50 and PLA/PBAT-75 are 62%, 46% and 68%, respectively. This result indicates that the degradation degree and rate of PLA/PBAT-50 are the lowest among the three formulations, which further demonstrates the negative correlation between the crystallinity and the degradation of the PLA/PBAT composite.

**Figure 7:**
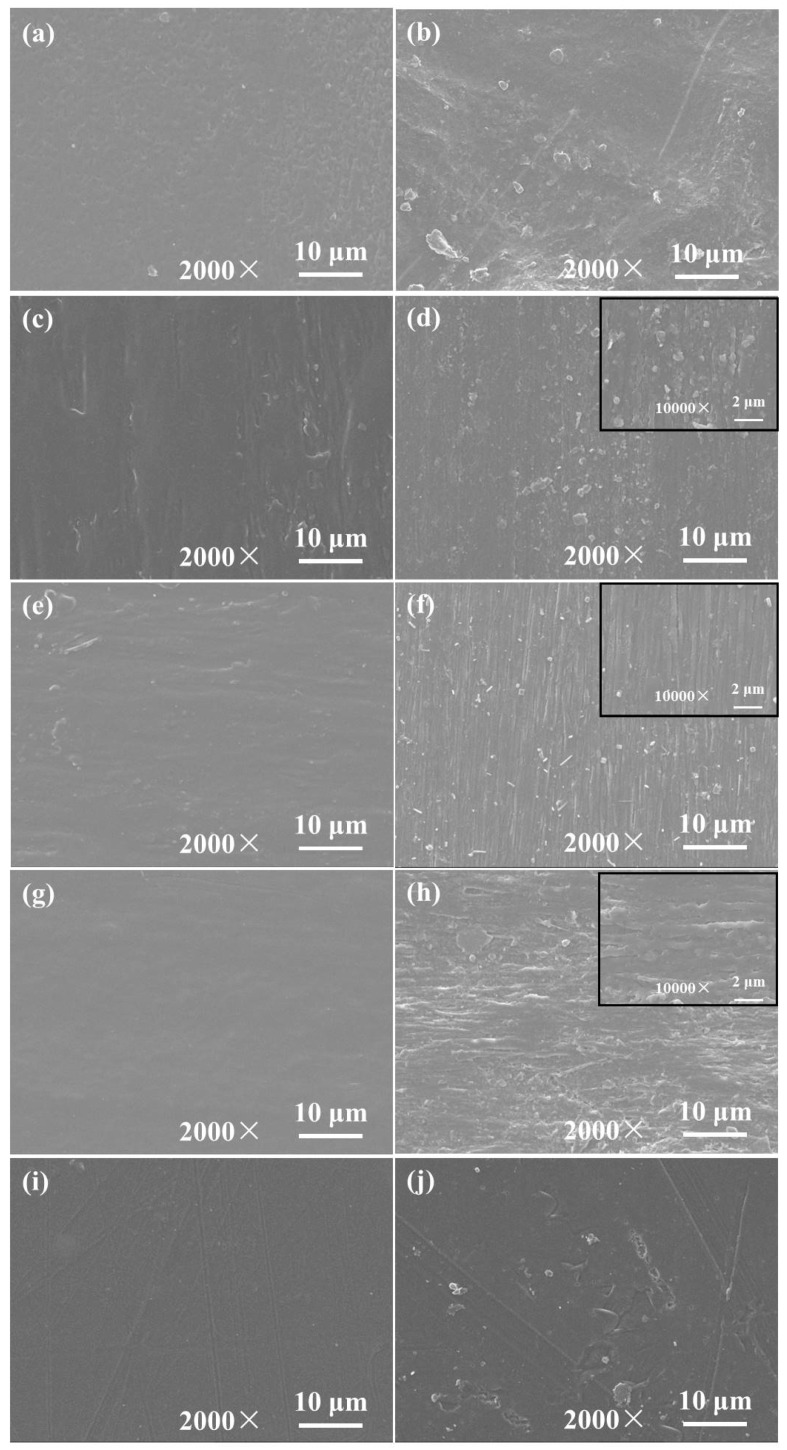
SEM micrographs of (**a**,**b**) PBAT, (**c**,**d**) PLA/PBAT-25, (**e**,**f**) PLA/PBAT-50, (**g**,**h**) PLA/PBAT-75 and (**i**,**j**) PLA before and after degradation for 24 months.

The morphology of immiscible polymer blends depends on the interaction between process conditions and blend components properties. When the dispersed phase concentration is well controlled, complex structures (such as ribbon-like, platelet, stratified and co-continuous structures) are formed during the flowing, which is basically controlled by factors such as the flow type and the intensity during its processing in the molten state, the viscosity ratio and the interfacial tension. The microstructure of blends before and after degradation were observed by SEM, as shown in Figure 7. The surface of pure PBAT is not so smooth before degradation, and after degradation numerous protrusions and cavities as well as eroded regions appeared in the surface of PBAT obviously. From the micro-morphologies of pure PLA in Figure 7i,j, except for minority impurities existing on the samples surface, there are no other defects in the surface before degradation. The surface shows many severe cracks after 24 months of degradation, which illustrates that PLA has degraded. The morphology of PLA/PBAT blends shown in Figure 7c–e reveal the elongated and fibrillar structure of the PLA dispersed phase in the blends arranged along the blowing direction. After 24 months of degradation, the amorphous regions in the blend samples with two phase morphology were eroded and formed a 3D porous network, which leads to the surfaces of blends having numerous protruding ribs and conspicuous gullies. Therefore, degradation had occurred in blends.

## 3. Experimental

### 3.1. Materials

Poly(lactic acid) (PLA) with the grade name of 2003D (l-lactic acid: 98.6%, d-lactic acid: 1.4%, a density of 1.24 g/cm^3^) was purchased from NatureWorks, Blair, NE, United States. The number-average molecular weight was 3.50 × 10^5^ g/mol, the polydispersity index was 2.55, $T_{g}$ = 61 °C, $T_{m}$ = 165 °C. The poly(butylene adipate-co-terephthalate) (PBAT) used in this work was Ecoflex FBX 7011 from BASF (Ludwigshafen, Germany) with the number-average molecular weight of 1.42 × 10^5^ g/mol, the polydispersity index was 2.46, $T_{g}$ = −29 °C, $T_{m}$ = 110–115 °C from BASF, Germany.

The muddy sediment and freshwater were collected beneath the low-water line of Baiyang Lake, Hebei. The sediment was conserved at 4 °C and was used within 4 weeks after sampling. The total organic carbon (TOC), pH and nitrogen content of the sediment were 2.7%, 7.4 and 0.1%, respectively.

### 3.2. Preparation of PLA/PBAT Films

Blends of PLA and PBAT with different weight ratios at 0/100, 25/75, 50/50, 75/25 and 100/0 were premixed in a high-speed mixer at 400 rpm for 5 min, followed with melt-extrusion via a co-rotating twin-screw extruder with a L/D ratio of 40 (PIE-35, Coperion Keya Machinery Co. Ltd., Nanjing, China). The blending was obtained at 190 °C with a screw speed of 90 rpm. PLA/PBAT films were prepared by extrusion blowing of blends with a single screw extruder (LP-S-50, LabTech Engineering Co Ltd., Samut Prakan, Thailand) at 190 °C. The films will be denoted as PLA, PBAT and PLA/PBAT-x, where x stands for the concentration of PLA.

### 3.3. Biodegradation of PLA/PBAT Films in Freshwater with Sediment

PLA/PBAT films were cut into 5 × 10 cm specimens for testing. The biodegradation testing of PLA/PBAT films with different formation was carried out respectively in tank with a size of 120 × 60 × 80 cm filled with 360 L freshwater and 4 kg sediment. The composite samples were buried flat 2 cm deep under the sediment surface. Three specimens of each sample were taken out every 6 months for testing.

### 3.4. Characterization

The FT-IR spectra of PLA/PBAT degradation samples were obtained with a Nicolet iS50 FT-IR spectrometer equipped with Scientific iD7 attenuated total reflection adjunct (Thermos Fisher scientific, Waltham, MA, USA) in a wavenumber range of 4000–400 cm^−1^. The thermal decomposition temperatures of samples were determined using a thermogravimetric analyzer, TA Universal V4.5A (TA Instruments, New Castle, DE, USA), from 40 to 500 °C with a heating rate of 20 °C/min under nitrogen atmosphere. The thermal properties of PLA/PBAT degradation samples were obtained by DSC with the instrument of Model Q100 (TA Instruments, New Castle, DE, USA).Samples weighing 5–10 mg were quickly heated to 180 °C, kept at a constant temperature for 3 min to eliminate the thermal history, followed by cooling to −50 °C at a rate of 20 °C/min in nitrogen atmosphere. The reheating of samples was carried out to 180 °C at a rate of 20 °C/min. Static water contact angles of the composites were measured by the sessile drop method using a 2 μL water droplet in a telescopic goniometer (OCA15 EC, Dataphysics, Filderstadt, Germany). The molecular weights of PLA/PBAT composites were determined by gel permeation chromatography (GPC) Waters 1515 Isocratic HPLC Pump (Waters, Milford, MA, USA), with the eluent of tetrahydrofuran at 30 °C. The film samples were rinsed off with deionized water and dried. Then, the specimens were treated by spray-gold and following this were observed using a scanning electron microscope (JOEL, JSM-7900F, Tokyo, Japan) with 5 kV acceleration voltage under different multiples. The chemical state of element on the film surface was investigated by X-ray photoelectron spectroscopy on a K-ALPHA^+^ (Thermos Fisher scientific, Waltham, MA, USA) with an Al K α X-ray source (1486.68 eV photons). To compensate for surface charging effects, all binding energies (BEs) were referenced to the C 1s hydrocarbon peak at 284.8 eV.

## 4. Conclusions

The biodegradation behavior of PBAT, PLA and PLA/PBAT blends in freshwater with sediment was affected by the content, aggregation structure, hydrophilia and biodegradation mechanisms of the components. The thermal stability, $T_{g}$ and molecular weight of PLA/PBAT composites decreased after the degradation. The crystallinity of PLA/PBAT composites increases with the degradation time, which is a result of the faster biodegradation of amorphous regions by microorganisms and enzymes. Elevated PLA content in composite leads to a higher increase in the O/C content ratio after degradation. After 24 months of degradation, the increase in the relative peak area proportion of C-O to C=O in PLA/PBAT-25, PLA/PBAT-50 and PLA/PBAT-75 is 62%, 46% and 68%, respectively. The lowest degradation degree and rate of PLA/PBAT-50 (among the three formulation) further demonstrated the negative correlation between crystallinity and degradation of PLA/PBAT composite. The biodegradation rates of PBAT and PLA in the PLA/PBAT blend were lower than those for the single materials.

## Figures and Tables

**Table 1:** The $X_{c}\left(\%\right)$ of PLA/PBAT with different component proportion before and after degradation.

	Before Degradation	After 6-Month Degradation	After 12-Month Degradation	After 24-Month Degradation
PLA/PBAT-25	2.5 ± 0.1	2.8 ± 0.2	3.4 ± 0.2	4.0 ± 0.2
PLA/PBAT-50	6.4 ± 0.3	6.7 ± 0.3	7.2 ± 0.4	7.4 ± 0.4
PLA/PBAT-75	1.4 ± 0.1	3.0 ± 0.2	3.4 ± 0.2	5.1 ± 0.3

**Table 2:** The element content of PLA/PBAT with different component proportion before and after degradation.

	Degradation Time	O 1s (Atomic %)	C 1s (Atomic %)	O/C Content Ratio
PBAT	before	26.03	70.33	0.37
	12 months	43.89	31.77	1.38
	24 months	43.84	30.42	1.44
PLA/PBAT-25	before	26.39	70.32	0.38
	12 months	27.47	62.84	0.44
	24 months	28.38	62.74	0.45
PLA/PBAT-50	before	26.94	68.29	0.39
	12 months	31.09	57.89	0.54
	24 months	33.2	54.41	0.61
PLA/PBAT-75	before	24.81	67.48	0.37
	12 months	28.38	60.84	0.47
	24 months	30.81	58.54	0.53
PLA	before	26.61	69.64	0.38
	12 months	35.8	50.55	0.71
	24 months	43.5	39.74	1.09

## Supplementary Materials

The following are available online. Figure S1: Static water contact angle of (a) PBAT, (b) PLA/PBAT-25, (c) PLA/PBAT-50, (d) PLA/PBAT-75, (e) PLA. Table S1: TGA of PLA/PBAT composite films before and degradating for 6 months, 12 months, 18 months and 24 months.
