# Supplementary material for: Biodegradation Behavior of Poly(Butylene Adipate-Co-Terephthalate) (PBAT), Poly(Lactic Acid) (PLA), and Their Blend in Freshwater with Sediment
Source: Molecules. 2020 Aug 29;25(17):3946. doi: 10.3390/molecules25173946 (PMC7504808; doi:10.3390/molecules25173946)
Supplement: Supplementary file 1 [file molecules-25-03946-s001.pdf]

Supplementary Materials

Biodegradation behavior of poly(butylene adipate-co-terephthalate) (PBAT), poly(lactic acid) (PLA), and their blend in freshwater sediment

Ye Fu [1], Gang Wu [2], Xinchao Bian [3], Jianbing Zeng [4], Yunxuan Weng [1, *]

1. Beijing Key Laboratory of Quality Evaluation Technology for Hygiene and Safety of Plastics, College of Chemistry and Materials Engineering, Beijing Technology and Business University, Beijing, 100037, China.
2. Collaborative Innovation Center for Eco-Friendly and Fire-Safety Polymeric Materials (MoE), National Engineering Laboratory of Eco-Friendly Polymeric Materials (Sichuan), State Key Laboratory of Polymer Materials Engineering, College of Chemistry, Sichuan University, Chengdu 610064, China.
3. Key Laboratory of Polymer Ecomaterials, Changchun Institute of Applied Chemistry, Chinese Academy of Sciences, Changchun, 130022, China.
4. Chongqing Key Laboratory of Soft-Matter Material Chemistry and Function Manufacturing, School of Chemistry and Chemical Engineering, Southwest University, Chongqing 400715, China.

* Corresponding Authors: Yunxuan Weng; Email: wyxuan@th.btbu.edu.cn

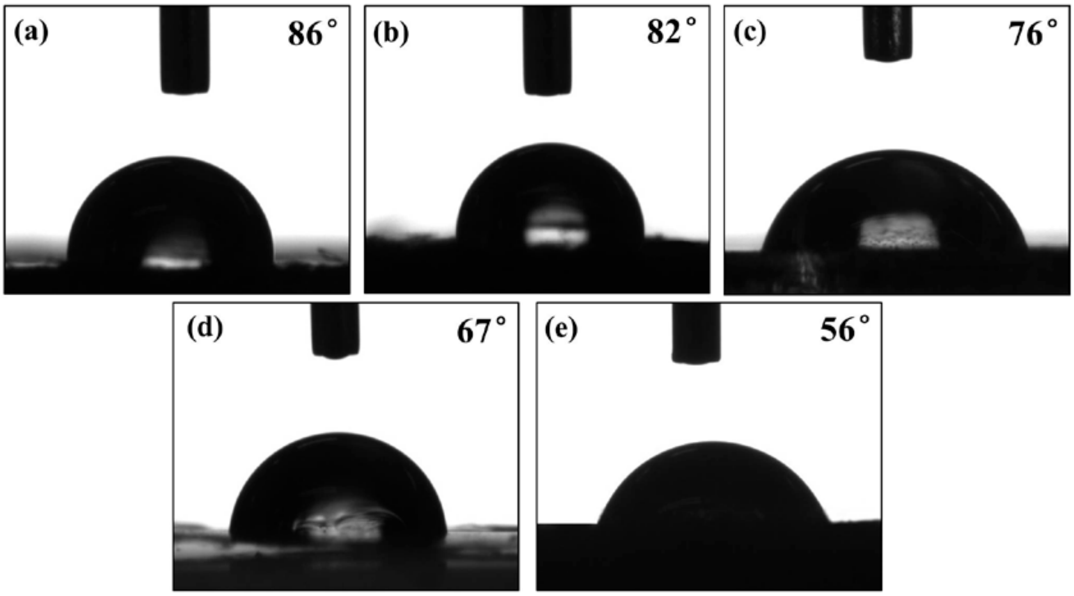

Fig S1 Static water contact angle of (a) PBAT, (b) PLA/PBAT-25, (c) PLA/PBAT-50, (d) PLA/PBAT-75, (e) PLA.

Table S1 TGA of PLA/PBAT composite films before and degradating for 6 months, 12 months, 18 months and 24 months.

Sample		Temperature at 5% Mass Loss (^{o}C)	Temperature at 50% Mass Loss (^{o}C)	Peak Degradation Temperature (^{o}C)
**PLA**	**0 month**	340.67	371.27	399.18
	**6 months**	312.79	362.54	392.69
	**12 months**	301.68	345.40	377.19
	**18 months**	295.57	331.63	363.68
	**24 months**	292.39	339.23	351.90
**PLA/PBAT-25**	**0 month**	336.03	387.26	487.69
	**6 months**	312.15	366.59	483.45
	**12 months**	317.03	367.13	480.05
	**18 months**	315.57	366.63	477.79
	**24 months**	307.85	359.44	475.03
**PLA/PBAT-50**	**0 month**	332.97	382.87	477.49
	**6 months**	304.23	369.81	473.49
	**12 months**	300.47	371.12	471.88
	**18 months**	297.79	370.96	471.03
	**24 months**	296.01	378.39	470.83
**PLA/PBAT-75**	**0 month**	330.30	371.12	473.91
	**6 months**	314.50	385.53	465.02
	**12 months**	284.96	369.37	462.77
	**18 months**	267.71	354.03	460.48
	**24 months**	283.92	379.28	473.08
**PBAT**	**0 month**	374.12	413.15	473.22
	**6 months**	356.28	417.58	457.12
	**12 months**	348.26	406.95	450.24
	**18 months**	341.26	412.21	462.94
	**24 months**	346.92	408.06	476.88
